# Author Correction: Exploiting interconnected synthetic lethal interactions between PARP inhibition and cancer cell reversible senescence

**DOI:** 10.1038/s41467-024-48270-9

**Published:** 2024-05-13

**Authors:** Hubert Fleury, Nicolas Malaquin, Véronique Tu, Sophie Gilbert, Aurélie Martinez, Marc-Alexandre Olivier, Skye Alexandre Sauriol, Laudine Communal, Kim Leclerc-Desaulniers, Euridice Carmona, Diane Provencher, Anne-Marie Mes-Masson, Francis Rodier

**Affiliations:** 1 Centre de recherche du Centre hospitalier de l’Université de Montréal (CRCHUM), Montreal, H2X 0A9 QC Canada; 2 Institut du cancer de Montréal, Montreal, H2X 0A9 QC Canada; 3 Division of Gynecologic Oncology, Université de Montréal, Montreal, H3C 3J7 QC Canada; 4 Department of Medicine, Université de Montréal, Montreal, H3C 3J7 QC Canada; 5 Department of Radiology, Radio-Oncology and Nuclear Medicine, Université de Montréal, Montreal, H3C 3J7 QC Canada

Correction to: *Nature Communications* 10.1038/s41467-019-10460-1, published online 11 June 2019

Since the publication of this work, Skye Alexandre Sauriol has changed their name from Alexandre Sauriol. This has now been amended in the HTML and PDF versions of the article.

