# Supplementary material for: Exploiting interconnected synthetic lethal interactions between PARP inhibition and cancer cell reversible senescence
Source: Nat Commun. 2019 Jun 11;10:2556. doi: 10.1038/s41467-019-10460-1 (PMC6560032; doi:10.1038/s41467-019-10460-1)
Supplement: Supplementary file 1 — Supplementary information [file 41467_2019_10460_MOESM1_ESM.pdf]

Exploiting interconnected synthetic lethal interactions between PARP inhibition and cancer cell reversible senescence

Fleury et al.

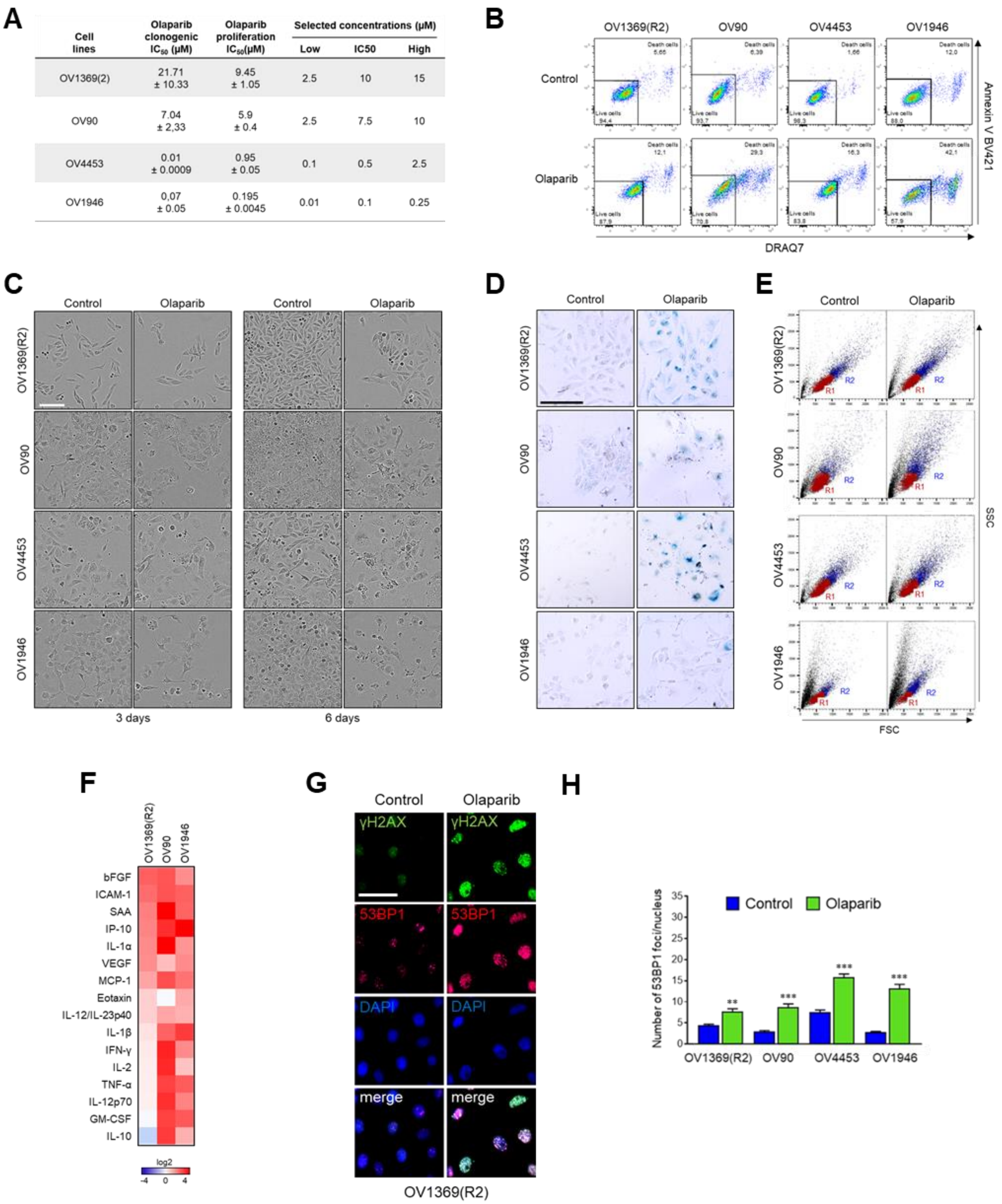

Cell lines	Olaparib clonogenic IC_{50} (µM)	Olaparib proliferation IC_{50}(µM)	Selected concentrations (µM) Low	IC50	High
OV1369(2)	21.71 ± 10.33	9.45 ± 1.05	2.5	10	15
OV90	7.04 ± 2,33	5.9 ± 0.4	2.5	7.5	10
OV4453	0.01 ± 0.0009	0.95 ± 0.05	0.1	0.5	2.5
OV1946	0,07 ± 0.05	0.195 ± 0.0045	0.01	0.1	0.25

Supplementary Figure 1. Olaparib induces a senescent phenotype in HGSOC cell lines.

A) Table of Olaparib IC_{50} (µM) displaying mean ± SEM data obtained by previous clonogenic assays[4] or by proliferation assays after 6 days of treatment. These data determined the low, IC_{50} and high concentrations used in this study. **B)** Representative flow cytometry analysis of apoptosis showing DRAQ7 positive cell population (X-axis) and AnnexinV positive cells (Y-axis) at day 6. Scale bar, 150µm. **C)** Images represent control and Olaparib-treated HGSOC cells at day 3 and 6 of Olaparib IC_{50} treatment. **D)** Representative morphology and SAβgal staining image for HGSOC cells treated

for 6 days with Olaparib IC_{50} concentration. Scale bar, 200µm. **E)** Representative flow cytometry analysis of the cell population plot for forward scatter factor (FSC, indicative of size, X-axis) and side scatter factor (SSC, indicative of granularity, Y-axis) (R2 (blue) =high FSC and SSC; R1 (red) = normal FSC and SSC) after 6 days of Olaparib IC_{50} concentration. **F)** Levels of secreted cytokines were measured by MSD serum based multiplex assay following 6 days treatment with Olaparib IC_{50} concentrations. **G)** Representative images of immunofluorescence of γH2AX (green) and 53BP-1 (Red) in OV1369(R2) cells untreated or treated 6 days with 5µM of Olaparib. Nuclei were counterstained with DAPI (bleu). Scale bar, 50µm. **H)** Number of 53BP1foci per nucleus in HGSOC cells lines treated with Olaparib IC_{50} concentrations for 6 days. Data in **(B-E, G)** are a representation of at least three independent experiments. For all the data, the mean ± SEM of three independent experiments is shown. Data were analyzed using the two-tail Student t-test. * Denotes $p < 0.05$, ** $p < 0.01$, and *** $p < 0.001$.

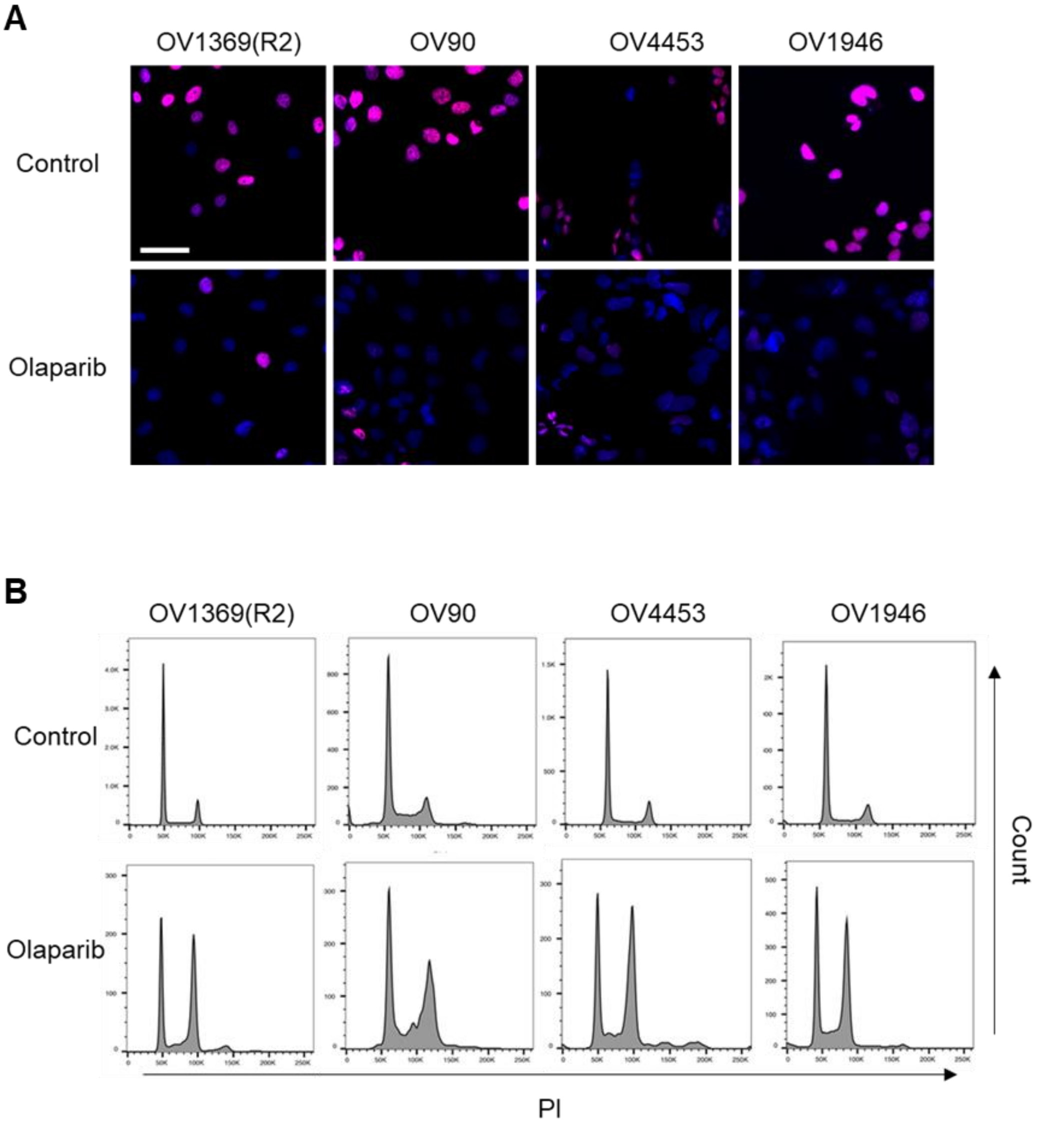

Supplementary Figure 2. Olaparib induces a stalled cell cycle in HGSOC cells.

A) Representative images of 8-hour EdU (Red) pulse after 6 days exposure of HGSOC cells to Olaparib IC_{50} concentrations. Nuclei were counterstained with DAPI (bleu). Scale bar, 50µm. **B)** Representative flow cytometry analysis of cell cycle showing PI levels (X-axis) and cell count (Y-axis) at day 6. Data in **(B)** are a representation of at least three independent experiments.

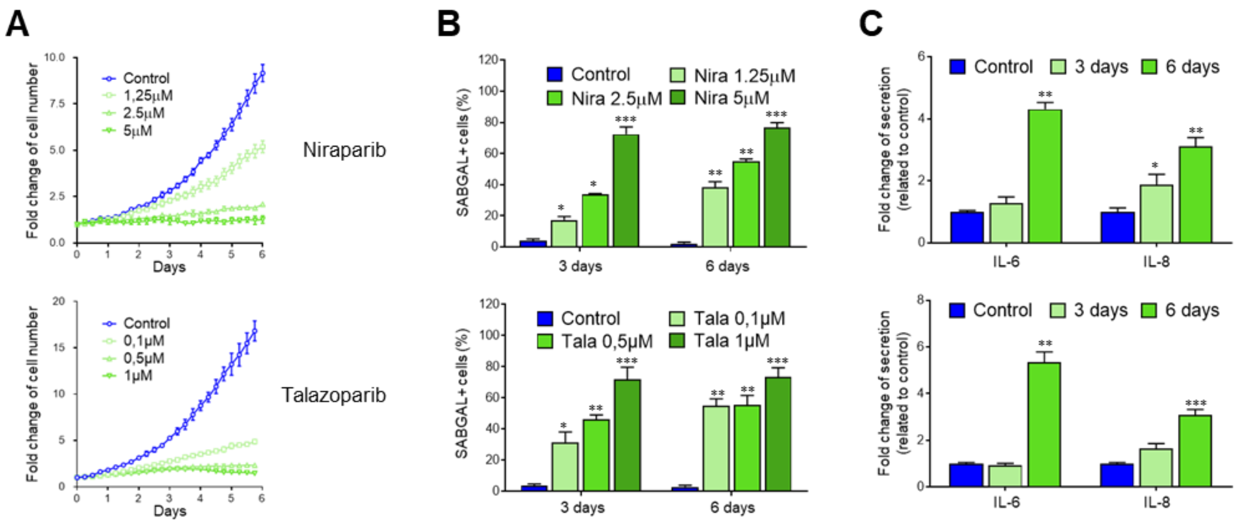

Supplementary Figure 3. Other PARPis also induce the senescence-like phenotype in HGSOC cells.

A) Cell proliferation curves were plotted with OV1369(R2) H2B-GFP cell line exposed to increasing concentrations of Niraparib or Talazoparib. **B-C)** Analyses of OV1369(R2) after 3 or 6 days in different concentrations of Niraparib and Talazoparib for levels of SA-β-gal positive cells **(B)** or 2.5µM Niraparib and 0.5µM Talazoparib for IL-6 and IL-8 levels **(C)**. Data in **(A)** are representative curves of at least three independent experiments. For all the data, the mean ± SEM of three independent experiments is shown. Data were analyzed using the two-tail Student t-test. * Denotes $p < 0.05$, ** $p < 0.01$, and *** $p < 0.001$

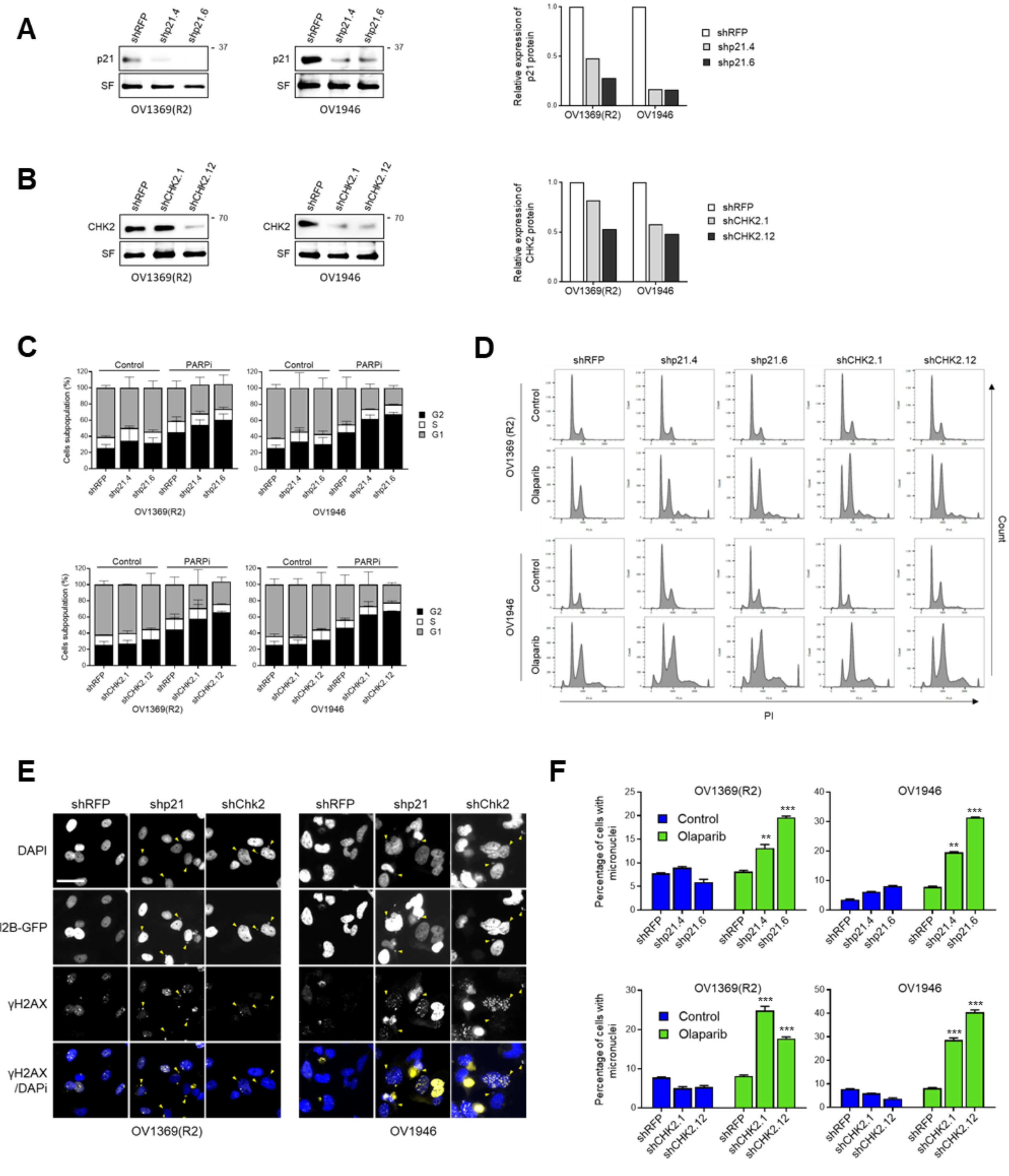

Supplementary Figure 4. Cell fate of HGSOC cells after p21 or Chk2 knockdown.

A-B) Western blot showing protein silencing by shRNA against p21 **(A)** or Chk2 **(B)** in HGSOC cells. **C)** Flow cytometry analysis of cell cycle populations following 6 days exposure of shp21 or shChk2 infected HGSOC cells to Olaparib IC_{50} concentrations. **D)** Representative flow cytometry analysis of DNA content (PI) following 6 days exposure of shp21 or shChk2 infected HGSOC cells to Olaparib IC_{50} concentrations. **E)** Representative images of γ-H2AX (yellow) immunofluorescence and micronuclei after 6 days of Olaparib IC_{50} treatment compared with non-treated controls. Yellow arrows represent micronuclei. Nuclei were counterstained with DAPI (bleu). Scale bar, 50µm. **F)**

Number of micronuclei per cells in shp21 or shChk2 infected HGSOC cells after 6 days of Olaparib IC_{50} were determined by analyzing >150 cells per condition. Data in **(A, D)** are a representation of at least three independent experiments. For all the data, the mean ± SEM of three independent experiments is shown. Data were analyzed using the two-tail Student t-test. * Denotes $p < 0.05$, ** $p < 0.01$, and *** $p < 0.001$.

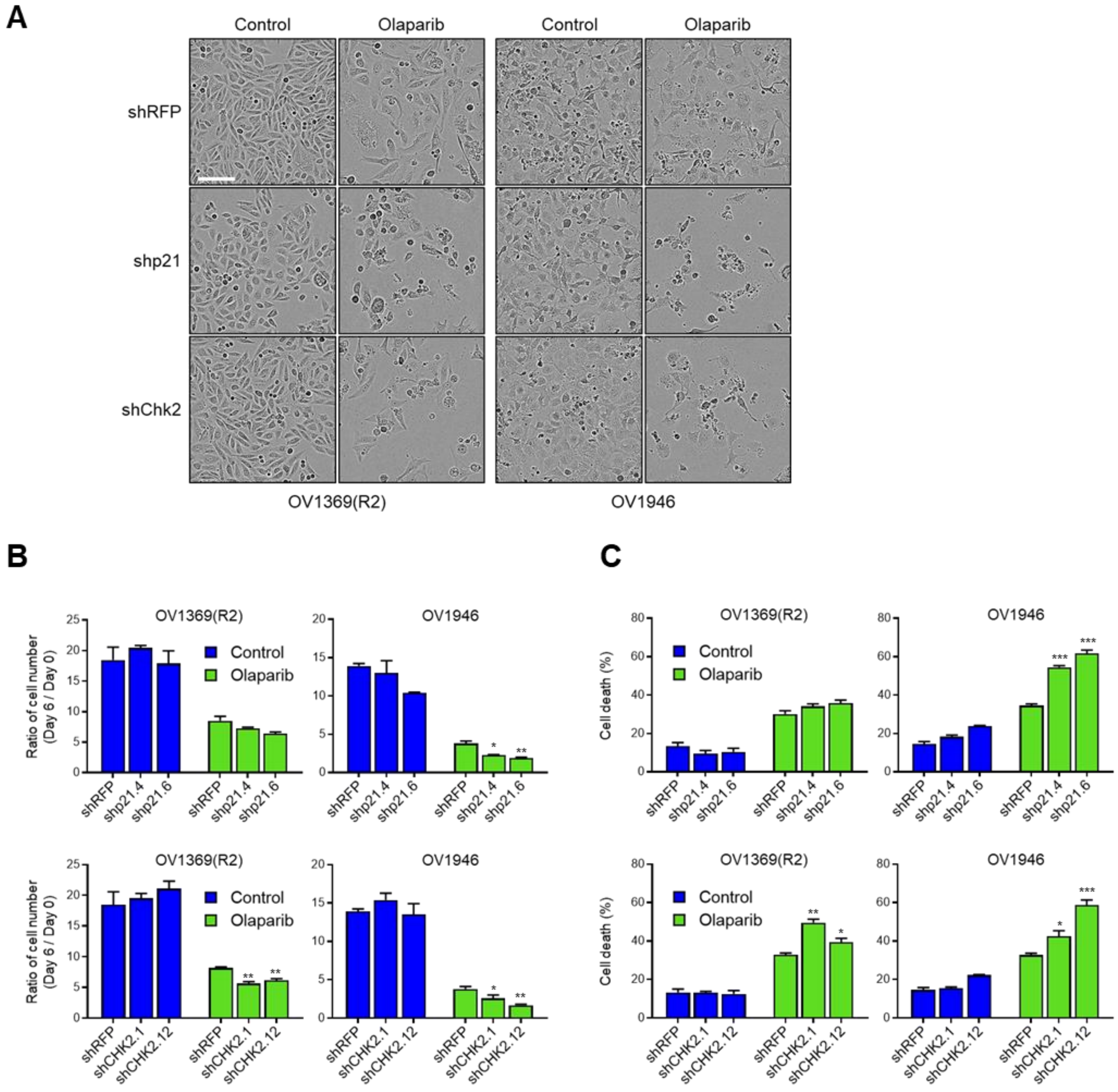

Supplementary Figure 5. Cell viability of HGSOC cells after p21 or Chk2 knockdown.

A) Representative images in phase-contrast microscopy of OV1369(R2) and OV1946 HGSOC cell lines, expressing shRFP, shp21 or shChk2, which were exposed to Olaparib IC_{50} concentrations for 6 days. Scale bar, 150µm. **B)** Fold change in cell number in shp21 or shChk2 infected HGSOC H2B-GFP cells treated to Olaparib IC_{50} concentrations. **C)** Percentage of dead cells analyzed by flow cytometry of shp21 or shChk2 infected HGSOC H2B-GFP cells treated to Olaparib IC_{50} concentrations. For all the data, the mean ± SEM of three independent experiments is shown. Data were analyzed using the two-tail Student t-test. * Denotes $p < 0.05$, ** $p < 0.01$, and *** $p < 0.001$

A

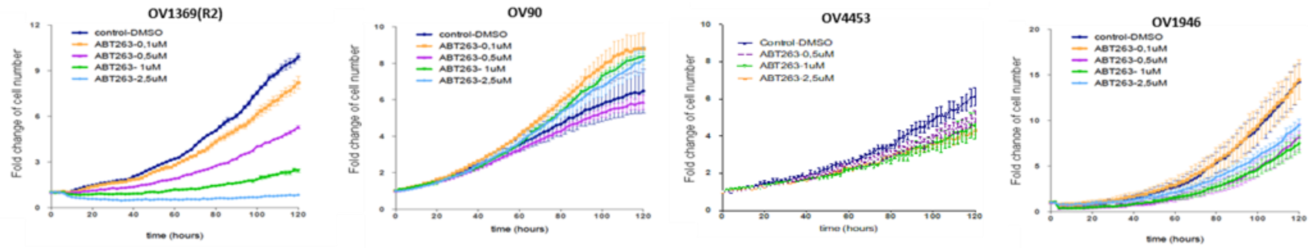

B

		OV1369(R2)		OV90		OV1946		OV4453	
		Olaparib	ABT-263	Olaparib	ABT-263	Olaparib	ABT-263	Olaparib	ABT-263
	Ratio	75	1	7.5	1	0.1	1	0.1	1
Concentration (µM)	D1	1	0.013	1	0.13	0.001	0.01	0.001	0.01
	D2	2.5	0.03	2.5	0.33	0.01	0.1	0.01	0.01
	D3	5	0.07	5	0.66	0.05	0.5	0.05	0.05
	D4	7.5	0.1	7.5	1	0.1	1	0.1	1
	D5	10	0.13	10	1.33	0.2	2	0.2	2
	D6	15	0.2	15	2	0.3	3	0.3	3
	D7	25	0.33	25	3.33	0.5	5	0.5	5

C

Name	Target	IC50 (µM)-OV1369(R2)	IC50 (µM)-OV1946
ABT-263	BCL-2; BCL-XL	0,34	2,58
ABT-199	BCL-2	4,38	6,06
A-1155463 (A115)	BCL-XL	1,94	27,7
A-1331852 (A133)	BCL-XL	0,07	4,2

D

OV1369(R2)

		ABT-263	ABT-199	A133	A115
Concentration (µM)	A1	0,05	0,5	0,01	0,025
	A2	0,1	1	0,025	0,05
	A3	0,25	2,5	0,05	0,1
	A4	0,5	5	0,075	0,5
	A5	0,75	10	0,1	1
	A6	1	20	0,5	2,5

OV1946

		ABT-263	ABT-199	A133	A115
Concentration (µM)	A1	0,25	0,5	0,5	0,5
	A2	0,5	1	1	1
	A3	0,75	2,5	2,5	2,5
	A4	1	5	5	5
	A5	2,5	10	10	10
	A6	5	20	20	20

Supplementary Figure 6. Bcl2/Bcl-XL inhibitors synergize with Olaparib in HGSOC cells.

A) Cell proliferation response of HGSOC cells to different concentrations of ABT-263 for 6 days with analysis of cell numbers every 6 hours. Control = non-treated. **B)** Table of Olaparib and ABT-263 doses (µM) used for the CI model study (constant ratio) for OV1369(R2), OV90, OV1946 and OV4453 cells. **C)** Table of ABT-263, ABT-199, A-1155463 and A-1331852 IC_{50} data obtained after 6 days treatment. **D)** Table of ABT-263, ABT-199, A-1155463 (A115) and A-1331852 (A133) doses (µM) used for the Bliss model study for the OV1369(R2) and OV1946 cell lines. Data in **(A,C)** are representative curves of at least three independent experiments.

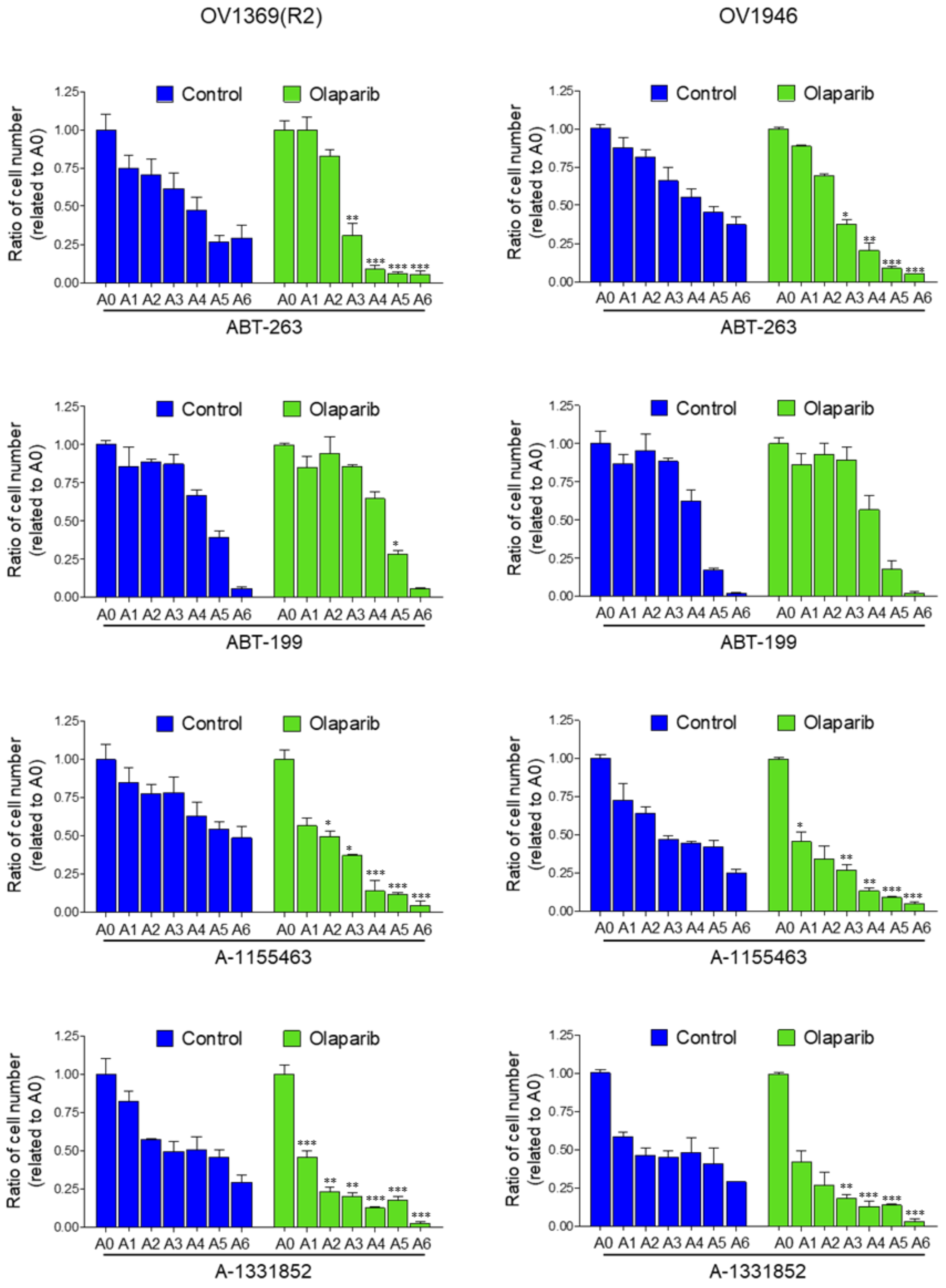

Supplementary Figure 7. Cell viability of Olaparib and distinct Bcl2/Bcl-XL inhibitors co-treatment.

Cells were co-treated with Olaparib IC_{50} concentrations and a range of ABT-263, ABT-199, A-1155463 or A-1331852 concentrations (see Supplementary Figure 6D) for 6 days. Bar graph shows mean ± SEM of the fold change in cell numbers on day 6 related to A0 (control non-treated for blue bars; Olaparib-treated for green bars) at different concentrations of Bcl2/Bcl-XL inhibitors added on day 3. For all the data, the mean ± SEM of three independent experiments is shown. Data were analyzed using the two-tail Student t-test. * Denotes $p < 0.05$, ** $p < 0.01$, and *** $p < 0.001$. Statistical analyses were performed between ABT-263 treated cells with and without Olaparib.

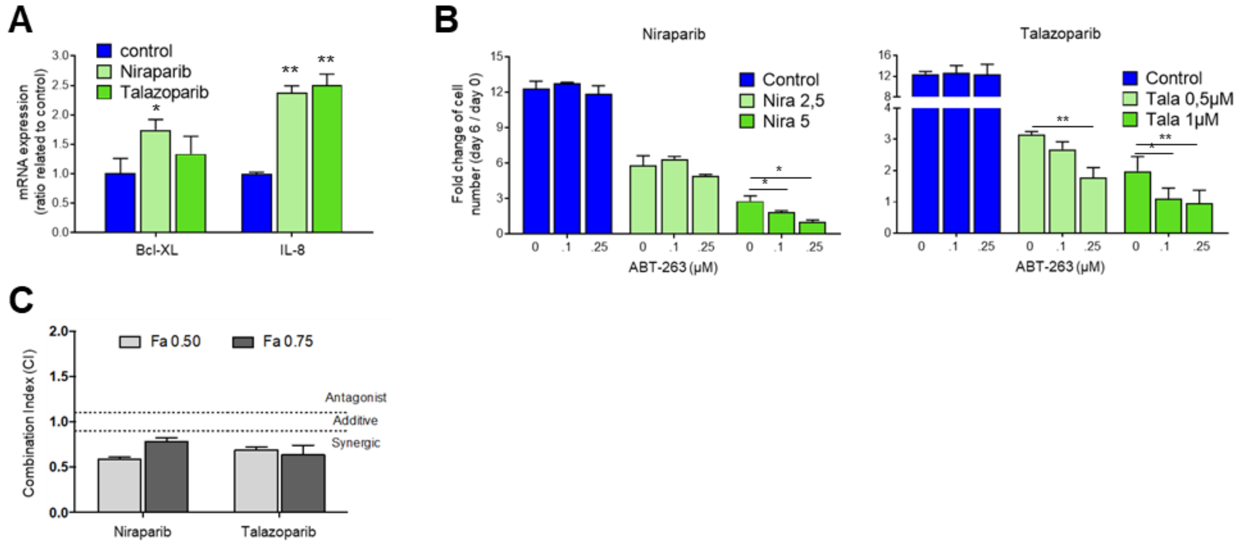

Supplementary Figure 8. Bcl2/Bcl-XL inhibitors synergize with other PARPis in HGSOC cells.

A) Relative mRNA levels of Bcl-XL and IL-8 evaluated by real-time Q-PCR in OV1369(R2) treated with 2.5µM Niraparib or 0.5µM Talazoparib for 6 days. **B)** Fold change in cell number for OV1369(R2) cells co-treated with Niraparib/ABT-263 or Talazoparib/ABT-263 for 6 days. **C)** Bar graph represents the CI at 0.50 and 0.75 fraction affected (Fa) of OV1369(R2) treated with Niraparib/ABT263 (10/1 ratio) or Talazoparib/ABT-263 (2/1 ratio). For all the data, the mean ± SEM of three independent experiments is shown. Data were analyzed using the two-tail Student t-test. * Denotes $p < 0.05$, ** $p < 0.01$, and *** $p < 0.001$

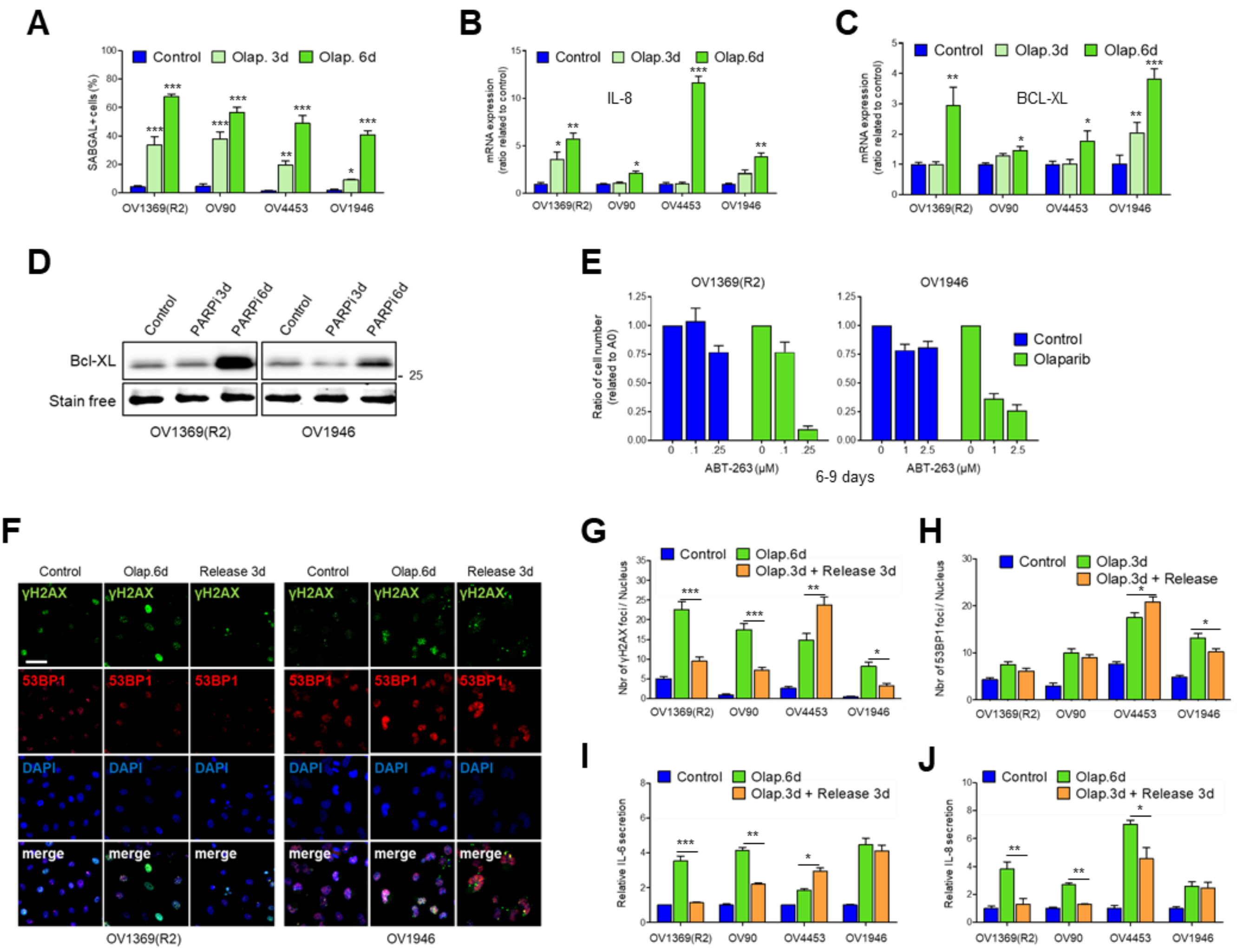

Supplementary Figure 9. Olaparib induces a gradual senescence phenotype targeted by senolytics.

A) SAβgal positive cells following exposure of HGSOC cells to Olaparib (Olap.) IC_{50} concentrations for 3 or 6 days. **B-C)** Real-time Q-PCR to evaluate the relative mRNA levels of IL-8 **(B)** and BCLXL **(C)** in HGSOC cells treated for 3 or 6 days with Olaparib IC_{50} concentration. Bars represent mean ± SEM of the fold change expression relative to control obtained in three independent experiments. **D)** Western blot detection of Bcl-XL on HGSOC cells treated with Olaparib for 3 or 6 days. **E)** OV1369(R2) and OV1946 cells were treated with Olaparib IC_{50} concentrations for 9 days with sequential addition of ABT-263 after 6 days of Olaparib. Bar graph shows mean ± SEM of the fold change in cell number related to A0 (control non-treated for blue bars; Olaparib-treated for green bars) at different ABT-263 concentrations. **F)** Representative images of γ-H2AX (green) and 53BP1 (red) immunostaining after 6 days Olaparib or 3 days Olaparib and then without drug for another 3 days of two selected cell lines [OV1369(R2) and OV1946]. Nuclei were counterstained with DAPI (bleu). Scale bar, 50µm. **G-H)** Cells under Olaparib IC_{50} treatment, the removal protocol and controls were stained for γ-H2AX **(G)** and 53BP1 **(H)** and analyzed by immunofluorescence imaging. **H-I)** Levels of IL-6 **(H)** and IL-8 **(I)** in HGSOC cells following treatment regimen of Fig.3B**.** Data in **(D)** are a representation of at least three independent experiments. For all the data, the mean ± SEM of three independent experiments is shown. Data were analyzed using the two-tail Student t-test. * Denotes $p < 0.05$, ** $p < 0.01$, and *** $p < 0.001$

A

Name	IC50 (µM)-OV1369(R2)	IC50 (µM)-OV1946
Piperlongumine (PPL)	0,58	1,48
Fisetin (F)	72,8	32,09
Dasatinib (D)	0,04	14,26
Quercitin (Q)	192,1	58,02

B

Concentration (µM)

OV1369(R2)

	PPL	Fisetin	Dasatinib	Quercetin
S1	0,1	1	0,0001	10
S2	0,25	5	0,0025	25
S3	0,5	10	0,005	50
S4	0,65	25	0,01	100
S5	0,75	75	0,1	200
S6	0,85	100	0,5	300

OV1946

	PPL	Fisetin	Dasatinib	Quercetin
S1	0,1	1	1	5
S2	0,25	5	2,5	10
S3	0,5	10	4	25
S4	0,65	25	7	50
S5	0,75	75	10	75
S6	0,85	100	15	100

C

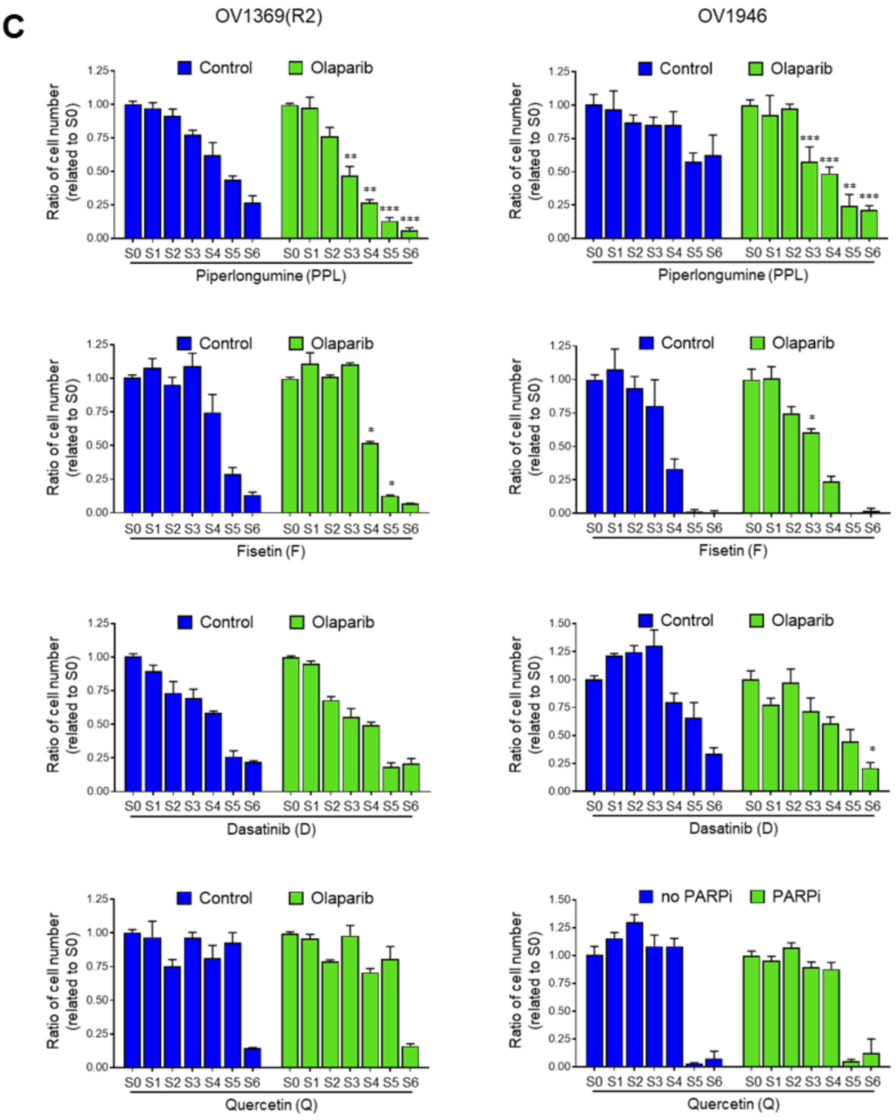

Supplementary Figure 10. Distinct senolytic drugs synergize with Olaparib in HGSOC cells.

A) Table of Piperlongumine (PPL), Fisetin (F), Dasatinib (D) and Quercetin (Q) IC_{50} (µM) displaying mean ± SEM data obtained after 6 days proliferation of OV1369(R2) and OV1946 cells; these data determined the range of concentrations used for this study. **B)** Table of PPL, F, D and Q doses (µM) used for combination treatments with Olaparib in OV1369(R2) and OV1946 cell lines. **C)** Cells were co-treated with Olaparib IC_{50} concentrations and a range of PPL, F, D or Q concentrations for 6 days. Bar graph shows mean ± SEM of the fold change in cell number related to S0 (control non-treated, blue bars; Olaparib-treated, green bars) at different concentrations of the senolytic drugs. For all the data, the mean ± SEM of three independent experiments is shown. Data were analyzed using the two-tail Student t-test. * Denotes $p < 0.05$, ** $p < 0.01$, and *** $p < 0.001$. Statistical analyses were performed between ABT-263 treated cells with and without Olaparib.

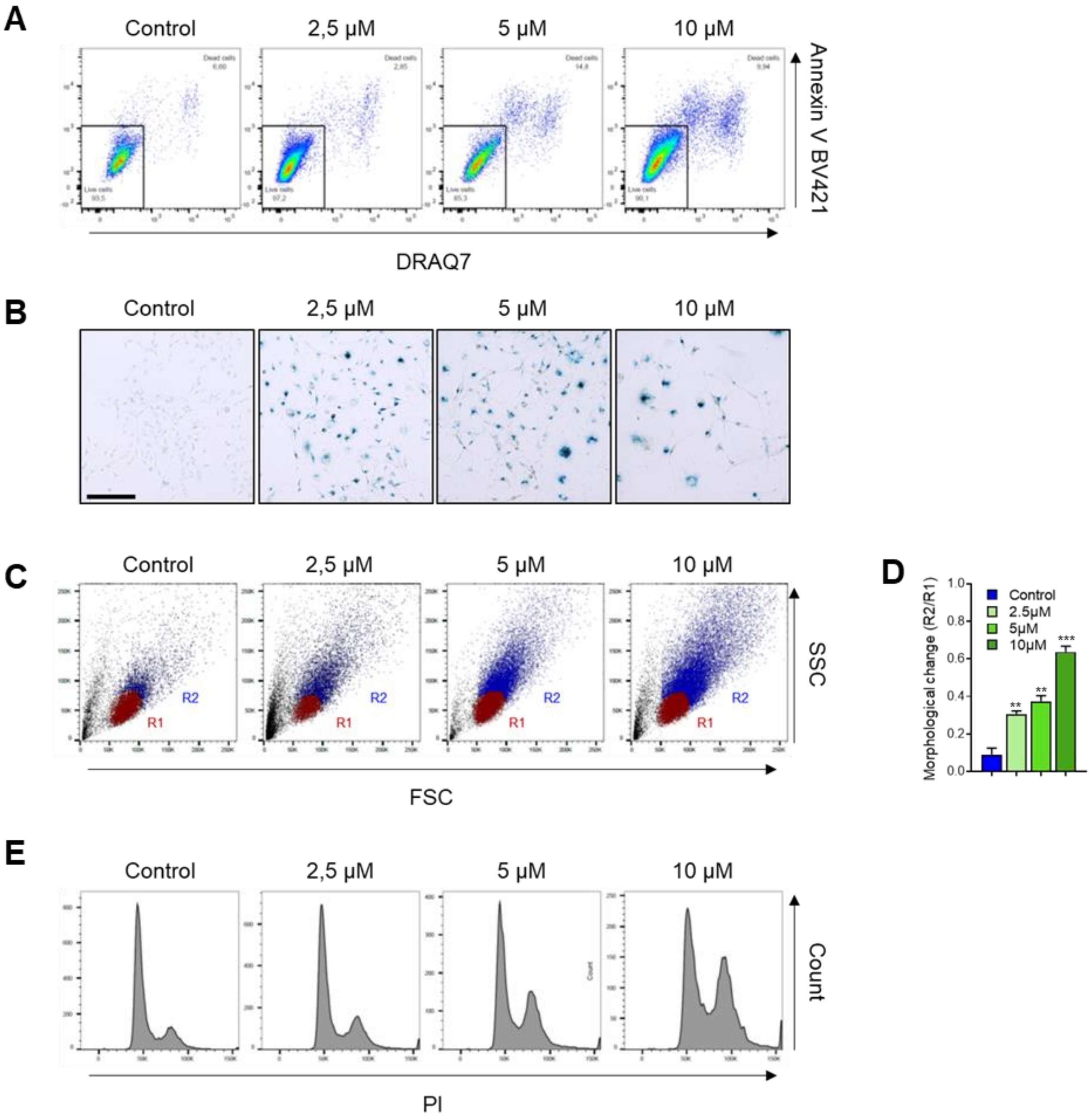

Supplementary Figure 11. Olaparib induces senescence-like phenotype in a TNBC cell line.

A-E) MDA-MB-231 cells were treated for 6 days with 2.5µM, 5µM or 10µM Olaparib. **A)** Representative flow cytometry analysis of apoptosis. **B)** Representative morphology and SAβgal staining images. Scale bar, 200µm. **C-D)** Representative flow cytometry analysis **(C)** and quantification **(D)** of the cell population plot for forward scatter factor (FSC, indicative of size, X-axis) and side scatter factor (SSC, indicative of granularity, Y-axis) (R2 (blue) =high FSC and SSC; R1 (red) = normal FSC and SSC). **E)** Representative flow cytometry analysis of cell cycle showing PI levels (X-axis) and cell count (Y-axis). Data in **(A-C, E)** are a representation of at least three independent experiments. For data in D, the mean ± SEM of three independent experiments is shown. Data were analyzed using the two-tail Student t-test. * Denotes $p < 0.05$, ** $p < 0.01$, and *** $p < 0.001$

A

MDA-MB-231

		ABT-263	A115	PPL
Concentration (µM)	S1	0,15625	0,078125	0,15625
	S2	0,3125	0,15625	0,3125
	S3	0,625	0,3125	0,625
	S4	1,25	0,625	1,25
	S5	2,5	1,25	2,5
	S6	5	2,5	5

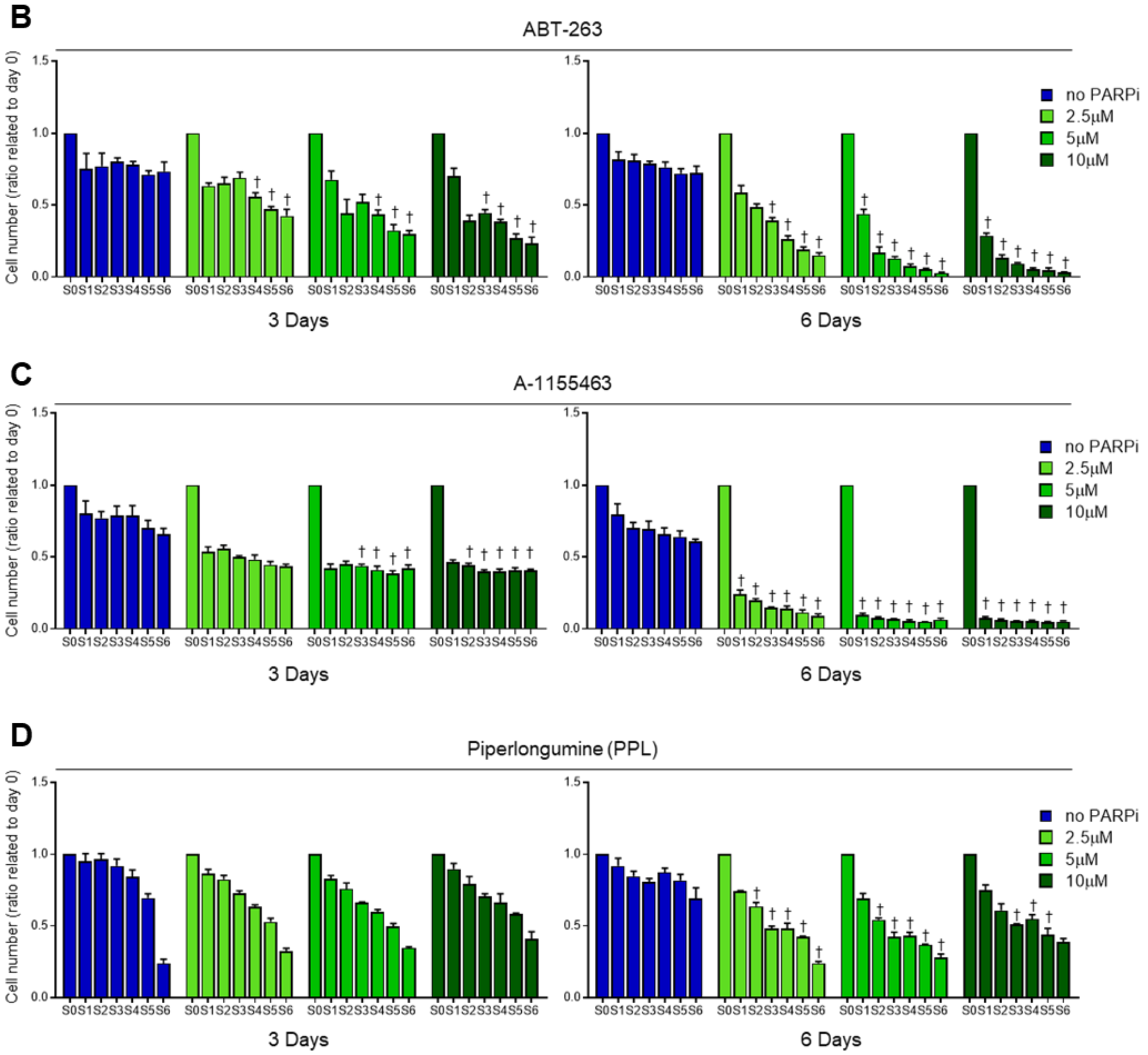

Supplementary Figure 12: Distinct senolytic drugs synergize with Olaparib in TNBC cell line.

A) Table of ABT-263, A-115463 and Piperlongumine (PPL) doses (µM) used for combination treatments with Olaparib in MDA-MB-231 cell lines. **B-D)** Cells were co-treated with Olaparib 2.5µM, 5µM and 10µM concentrations and a range of ABT-263 **(B)**, A-1155463 **(C)** and PPL **(D)** concentrations for 6 days. Bar graph shows mean ± SEM of the fold change in cell number related to day 0 (no PARP: blue bars; Olaparib-treated, green bars) at different concentrations of Olaparib and senolytic drugs. For all the data, the mean ± SEM of three independent experiments is shown. Data were analyzed using the two-tail Student t-test. † Denotes $p < 0.01$. Statistical analyses were performed between ABT-263 treated cells with and without Olaparib.

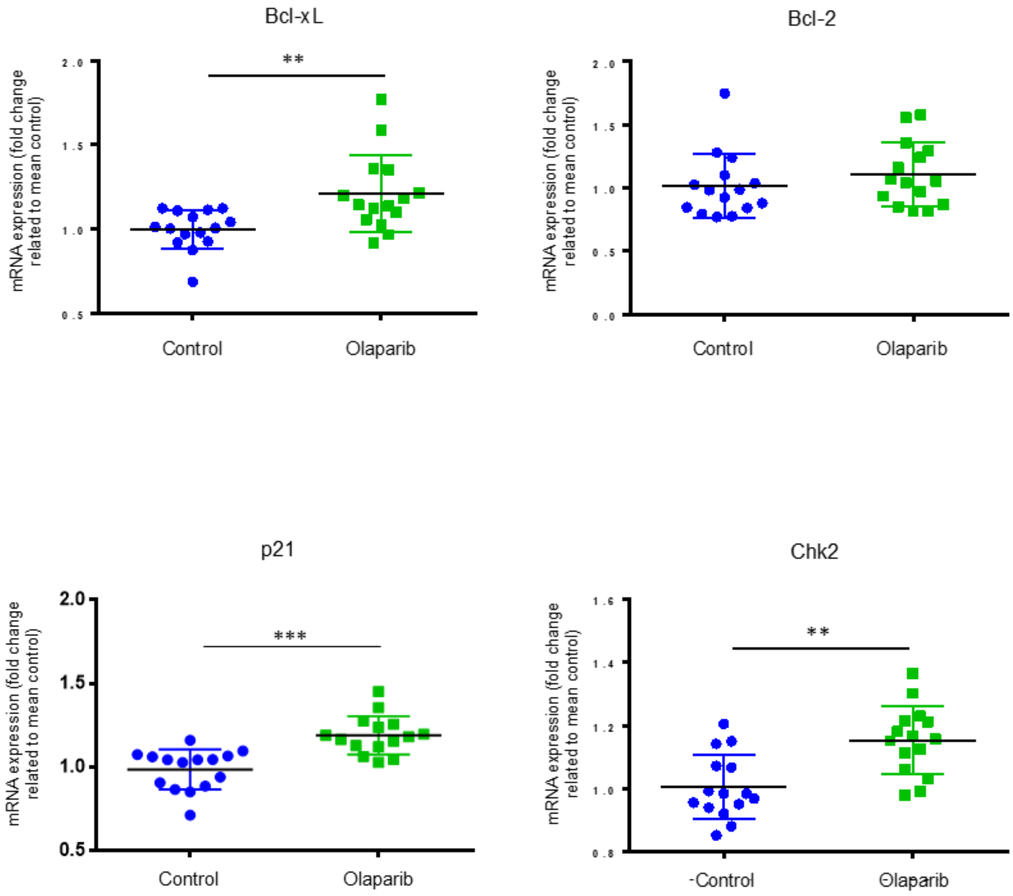

Supplementary Figure 13: Gene expression analysis of selected Olaparib-induced senescent markers on MDA-MB-231 xenograft tumors.

Q-PCR was performed to determine the expression levels of BCL-XL, BCL2, p21 and CHK2 in xenograft tumor tissues harvested from mice treated with Olaparib or vehicle for 12 days (each data point correspond to one tumor, n=15 for each group). For all the data, the mean ± SEM of three independent experiments is shown. Data were analyzed using the two-tail Student t-test. * Denotes $p < 0.05$, ** $p < 0.01$, and *** $p < 0.001$.

Figure 4I

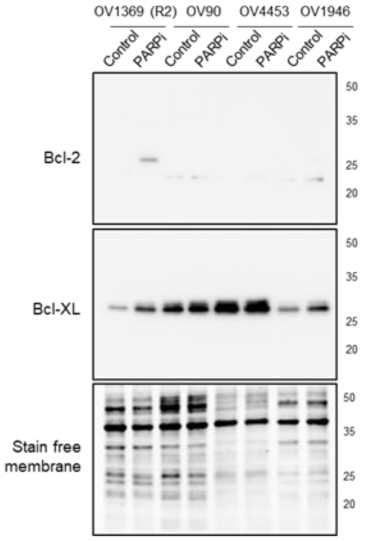

Figure 6G

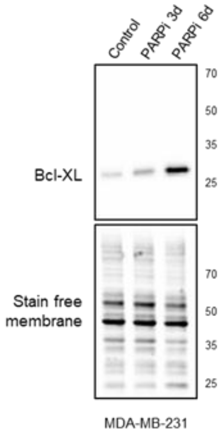

Supplementary Figure 4A

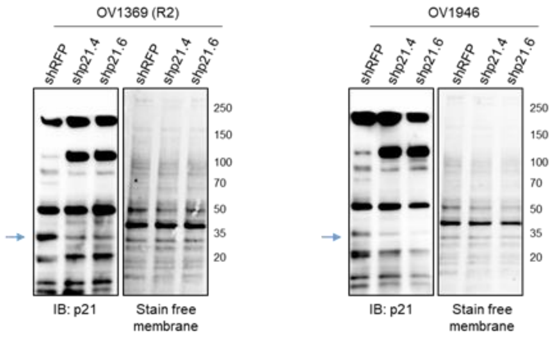

Supplementary Figure 4B

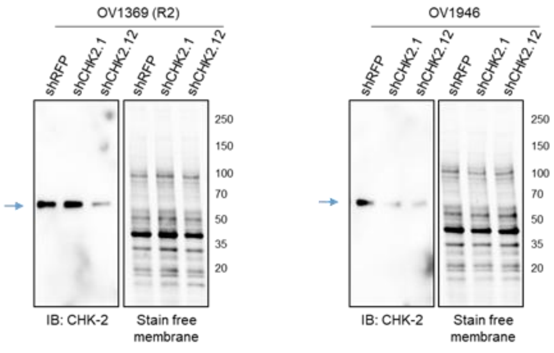

Supplementary Figure 9D

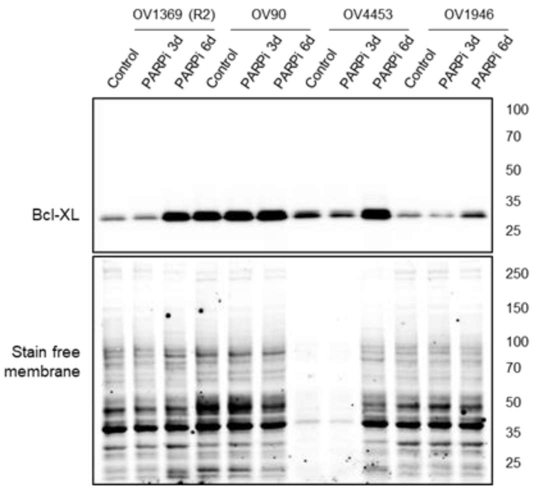

Supplementary Figure 14: Original image of western blots and stain free membranes.

OV4453 2-way Anova test with Bonferroni post-test corrections

Bonferroni's multiple comparisons test	95,00% CI of diff,	Significant?	Summary	Adjusted P Value
Row 1 day 0				
Control vs. Olaparib	-0,2573 to 0,2573	No	ns	>0,9999
Control vs. ABT263	-0,2540 to 0,2540	No	ns	>0,9999
Control vs. Combo	-0,2573 to 0,2573	No	ns	>0,9999
Olaparib vs. ABT263	-0,2265 to 0,2265	No	ns	>0,9999
Olaparib vs. Combo	-0,2301 to 0,2301	No	ns	>0,9999
ABT263 vs. Combo	-0,2265 to 0,2265	No	ns	>0,9999
Row 2 day 3				
Control vs. Olaparib	-0,1843 to 0,3612	No	ns	>0,9999
Control vs. ABT263	-0,2308 to 0,2773	No	ns	>0,9999
Control vs. Combo	-0,1979 to 0,3166	No	ns	>0,9999
Olaparib vs. ABT263	-0,3091 to 0,1787	No	ns	>0,9999
Olaparib vs. Combo	-0,2764 to 0,2182	No	ns	>0,9999
ABT263 vs. Combo	-0,1904 to 0,2626	No	ns	>0,9999
Row 3 day 7				
Control vs. Olaparib	-0,1213 to 0,3933	No	ns	0,9697
Control vs. ABT263	-0,05202 to 0,4560	No	ns	0,2134
Control vs. Combo	0,002082 to 0,5166	Yes	*	0,047
Olaparib vs. ABT263	-0,1605 to 0,2925	No	ns	>0,9999
Olaparib vs. Combo	-0,1068 to 0,3534	No	ns	0,9352
ABT263 vs. Combo	-0,1691 to 0,2838	No	ns	>0,9999
Row 4 day 10				
Control vs. Olaparib	-0,1271 to 0,3989	No	ns	>0,9999
Control vs. ABT263	-0,04742 to 0,4722	No	ns	0,1846
Control vs. Combo	0,004786 to 0,5549	Yes	*	0,0437
Olaparib vs. ABT263	-0,1500 to 0,3030	No	ns	>0,9999
Olaparib vs. Combo	-0,09985 to 0,3877	No	ns	0,7088
ABT263 vs. Combo	-0,1729 to 0,3078	No	ns	>0,9999
Row 5 day 14				
Control vs. Olaparib	0,07380 to 0,5997	Yes	**	0,0045
Control vs. ABT263	0,1761 to 0,7092	Yes	****	<0,0001
Control vs. Combo	0,3367 to 0,8868	Yes	****	<0,0001
Olaparib vs. ABT263	-0,1283 to 0,3401	No	ns	>0,9999
Olaparib vs. Combo	0,03118 to 0,5188	Yes	*	0,0178
ABT263 vs. Combo	-0,07859 to 0,4167	No	ns	0,4258
Row 6 day 18				
Control vs. Olaparib	0,03010 to 0,6623	Yes	*	0,0234
Control vs. ABT263	0,1350 to 0,7515	Yes	***	0,001
Control vs. Combo	0,4355 to 1,088	Yes	****	<0,0001
Olaparib vs. ABT263	-0,1578 to 0,3518	No	ns	>0,9999
Olaparib vs. Combo	0,1392 to 0,6922	Yes	***	0,0005
ABT263 vs. Combo	0,05119 to 0,5861	Yes	*	0,0102
Row 7 day 22				
Control vs. Olaparib	0,1741 to 0,8063	Yes	***	0,0003
Control vs. ABT263	0,2773 to 0,8887	Yes	****	<0,0001
Control vs. Combo	0,5798 to 1,246	Yes	****	<0,0001
Olaparib vs. ABT263	-0,1590 to 0,3446	No	ns	>0,9999
Olaparib vs. Combo	0,1384 to 0,7068	Yes	***	0,0006
ABT263 vs. Combo	0,05716 to 0,6024	Yes	**	0,0087

Supplementary Table 1

OV1946 2-way Anova test with Bonferroni post-test corrections

Bonferroni's multiple comparisons test	95,00% CI of diff,	Significant?	Summary	Adjusted P Value
Row 1				
Control vs. Olaparib	-0,2615 to 0,2615	No	ns	>0,9999
Control vs. ABT-263	-0,2743 to 0,2743	No	ns	>0,9999
Control vs. Combo	-0,2652 to 0,2652	No	ns	>0,9999
Olaparib vs. ABT-263	-0,2481 to 0,2481	No	ns	>0,9999
Olaparib vs. Combo	-0,2380 to 0,2380	No	ns	>0,9999
ABT-263 vs. Combo	-0,2520 to 0,2520	No	ns	>0,9999
Row 2				
Control vs. Olaparib	-0,1645 to 0,3585	No	ns	>0,9999
Control vs. ABT-263	-0,3344 to 0,2141	No	ns	>0,9999
Control vs. Combo	-0,1648 to 0,3656	No	ns	>0,9999
Olaparib vs. ABT-263	-0,4052 to 0,09091	No	ns	0,5621
Olaparib vs. Combo	-0,2346 to 0,2415	No	ns	>0,9999
ABT-263 vs. Combo	-0,09139 to 0,4126	No	ns	0,5505
Row 3				
Control vs. Olaparib	-0,07042 to 0,4579	No	ns	0,3148
Control vs. ABT-263	-0,3198 to 0,2288	No	ns	>0,9999
Control vs. Combo	-0,06820 to 0,4622	No	ns	0,2972
Olaparib vs. ABT-263	-0,4901 to 0,01164	No	ns	0,071
Olaparib vs. Combo	-0,2377 to 0,2442	No	ns	>0,9999
ABT-263 vs. Combo	-0,009482 to 0,4945	No	ns	0,0665
Row 4				
Control vs. Olaparib	-0,1093 to 0,4190	No	ns	0,7244
Control vs. ABT-263	-0,3449 to 0,2036	No	ns	>0,9999
Control vs. Combo	-0,03849 to 0,4919	No	ns	0,1436
Olaparib vs. ABT-263	-0,4764 to 0,02533	No	ns	0,1056
Olaparib vs. Combo	-0,1691 to 0,3128	No	ns	>0,9999
ABT-263 vs. Combo	0,04540 to 0,5494	Yes	*	0,0113
Row 5				
Control vs. Olaparib	0,04687 to 0,5752	Yes	*	0,0116
Control vs. ABT-263	-0,2793 to 0,2693	No	ns	>0,9999
Control vs. Combo	0,2477 to 0,7781	Yes	****	<0,0001
Olaparib vs. ABT-263	-0,5669 to -0,06514	Yes	**	0,0055
Olaparib vs. Combo	-0,03913 to 0,4428	No	ns	0,1614
ABT-263 vs. Combo	0,2659 to 0,7698	Yes	****	<0,0001
Row 6				
Control vs. Olaparib	0,02560 to 0,6055	Yes	*	0,0247
Control vs. ABT-263	-0,3692 to 0,2428	No	ns	>0,9999
Control vs. Combo	0,2227 to 0,8192	Yes	****	<0,0001
Olaparib vs. ABT-263	-0,6495 to -0,1080	Yes	**	0,0014
Olaparib vs. Combo	-0,05652 to 0,4674	No	ns	0,2291
ABT-263 vs. Combo	0,3046 to 0,8638	Yes	****	<0,0001
Row 7				
Control vs. Olaparib	0,03929 to 0,6192	Yes	*	0,0166
Control vs. ABT-263	-0,2530 to 0,3590	No	ns	>0,9999
Control vs. Combo	0,3156 to 0,9121	Yes	****	<0,0001
Olaparib vs. ABT-263	-0,5470 to -0,005538	Yes	*	0,0427
Olaparib vs. Combo	0,02265 to 0,5465	Yes	*	0,0251
ABT-263 vs. Combo	0,2812 to 0,8405	Yes	****	<0,0001

Supplementary Table 2

MDA-MB-231 2-way Anova test with Bonferroni post-test corrections

Bonferroni's multiple comparisons test	95,00% CI of diff,	Significant?	Summary	Adjusted P Value
Row 1				
Control vs. Olaparib	-105,0 to 40,93	No	ns	>0,9999
Control vs. ABT-263	-81,47 to 64,48	No	ns	>0,9999
Control vs. Olaparib + ABT-263	-103,9 to 42,07	No	ns	>0,9999
Olaparib vs. ABT-263	-48,16 to 95,25	No	ns	>0,9999
Olaparib vs. Olaparib + ABT-263	-70,57 to 72,85	No	ns	>0,9999
ABT-263 vs. Olaparib + ABT-263	-94,11 to 49,30	No	ns	>0,9999
Row 2				
Control vs. Olaparib	-38,82 to 107,1	No	ns	>0,9999
Control vs. ABT-263	-68,99 to 76,96	No	ns	>0,9999
Control vs. Olaparib + ABT-263	-70,89 to 75,06	No	ns	>0,9999
Olaparib vs. ABT-263	-101,9 to 41,53	No	ns	>0,9999
Olaparib vs. Olaparib + ABT-263	-103,8 to 39,63	No	ns	>0,9999
ABT-263 vs. Olaparib + ABT-263	-73,61 to 69,81	No	ns	>0,9999
Row 3				
Control vs. Olaparib	-38,41 to 107,5	No	ns	>0,9999
Control vs. ABT-263	-53,85 to 92,10	No	ns	>0,9999
Control vs. Olaparib + ABT-263	-52,89 to 93,06	No	ns	>0,9999
Olaparib vs. ABT-263	-87,15 to 56,27	No	ns	>0,9999
Olaparib vs. Olaparib + ABT-263	-86,19 to 57,23	No	ns	>0,9999
ABT-263 vs. Olaparib + ABT-263	-70,75 to 72,67	No	ns	>0,9999
Row 4				
Control vs. Olaparib	-40,37 to 105,6	No	ns	>0,9999
Control vs. ABT-263	-62,39 to 83,56	No	ns	>0,9999
Control vs. Olaparib + ABT-263	-42,78 to 103,2	No	ns	>0,9999
Olaparib vs. ABT-263	-93,73 to 49,69	No	ns	>0,9999
Olaparib vs. Olaparib + ABT-263	-74,11 to 69,30	No	ns	>0,9999
ABT-263 vs. Olaparib + ABT-263	-52,09 to 91,32	No	ns	>0,9999
Row 5				
Control vs. Olaparib	27,74 to 173,7	Yes	**	0,0017
Control vs. ABT-263	-44,90 to 101,0	No	ns	>0,9999
Control vs. Olaparib + ABT-263	68,76 to 214,7	Yes	****	<0,0001
Olaparib vs. ABT-263	-144,3 to -0,9342	Yes	*	0,0452
Olaparib vs. Olaparib + ABT-263	-30,68 to 112,7	No	ns	0,78
ABT-263 vs. Olaparib + ABT-263	41,96 to 185,4	Yes	***	0,0002
Row 6				
Control vs. Olaparib	-0,007868 to 145,9	No	ns	0,05
Control vs. ABT-263	-87,57 to 58,38	No	ns	>0,9999
Control vs. Olaparib + ABT-263	84,85 to 230,8	Yes	****	<0,0001
Olaparib vs. ABT-263	-159,3 to -15,86	Yes	**	0,0078
Olaparib vs. Olaparib + ABT-263	13,15 to 156,6	Yes	*	0,011
ABT-263 vs. Olaparib + ABT-263	100,7 to 244,1	Yes	****	<0,0001
Row 7				
Control vs. Olaparib	27,40 to 173,3	Yes	**	0,0018
Control vs. ABT-263	-75,76 to 70,19	No	ns	>0,9999
Control vs. Olaparib + ABT-263	126,1 to 272,0	Yes	****	<0,0001
Olaparib vs. ABT-263	-174,9 to -31,45	Yes	***	0,001
Olaparib vs. Olaparib + ABT-263	26,96 to 170,4	Yes	**	0,0018
ABT-263 vs. Olaparib + ABT-263	130,1 to 273,5	Yes	****	<0,0001

Supplementary Table 3
