# Supplementary material for: Exploiting interconnected synthetic lethal interactions between PARP inhibition and cancer cell reversible senescence
Source: Nat Commun. 2019 Jun 11;10:2556. doi: 10.1038/s41467-019-10460-1 (PMC6560032; doi:10.1038/s41467-019-10460-1)
Supplement: Supplementary file 3 — Reporting Summary [file 41467_2019_10460_MOESM3_ESM.pdf]

nature research

Corresponding author(s): DBPR - NCOMMS-18-14775

Last updated by author(s): Apr 30, 2019

Reporting Summary

Nature Research wishes to improve the reproducibility of the work that we publish. This form provides structure for consistency and transparency in reporting. For further information on Nature Research policies, see Authors & Referees and the Editorial Policy Checklist.

Statistics

For all statistical analyses, confirm that the following items are present in the figure legend, table legend, main text, or Methods section.

n/a	Confirmed	
☐	☒	The exact sample size (*n*) for each experimental group/condition, given as a discrete number and unit of measurement
☐	☒	A statement on whether measurements were taken from distinct samples or whether the same sample was measured repeatedly
☐	☒	The statistical test(s) used AND whether they are one- or two-sided<br>*Only common tests should be described solely by name; describe more complex techniques in the Methods section.*
☒	☐	A description of all covariates tested
☐	☒	A description of any assumptions or corrections, such as tests of normality and adjustment for multiple comparisons
☐	☒	A full description of the statistical parameters including central tendency (e.g. means) or other basic estimates (e.g. regression coefficient) AND variation (e.g. standard deviation) or associated estimates of uncertainty (e.g. confidence intervals)
☒	☐	For null hypothesis testing, the test statistic (e.g. *F*, *t*, *r*) with confidence intervals, effect sizes, degrees of freedom and *P* value noted<br>*Give P values as exact values whenever suitable.*
☒	☐	For Bayesian analysis, information on the choice of priors and Markov chain Monte Carlo settings
☒	☐	For hierarchical and complex designs, identification of the appropriate level for tests and full reporting of outcomes
☒	☐	Estimates of effect sizes (e.g. Cohen's *d*, Pearson's *r*), indicating how they were calculated

Our web collection on statistics for biologists contains articles on many of the points above.

Software and code

Policy information about availability of computer code

Data collection	TCGA- Ovarian Serous Cystadenocarcinoma (TCGA-Provisional) U133-Expression-Microarray-cBioportal
Data analysis	graph pad 8.1, Flow jo, axiovision 4.9, Compusyn

For manuscripts utilizing custom algorithms or software that are central to the research but not yet described in published literature, software must be made available to editors/reviewers. We strongly encourage code deposition in a community repository (e.g. GitHub). See the Nature Research guidelines for submitting code & software for further information.

Data

Policy information about availability of data

All manuscripts must include a data availability statement. This statement should provide the following information, where applicable:

- Accession codes, unique identifiers, or web links for publicly available datasets
- A list of figures that have associated raw data
- A description of any restrictions on data availability

TThe authors declare that the data supporting the findings of this study are available within the paper and its supplementary information files. If needed, additional information is available from the corresponding author upon reasonable request.

Field-specific reporting

Please select the one below that is the best fit for your research. If you are not sure, read the appropriate sections before making your selection.

☒ Life sciences ☐ Behavioural & social sciences ☐ Ecological, evolutionary & environmental sciences

Life sciences study design

All studies must disclose on these points even when the disclosure is negative.

Sample size	The chosen sample size are based on the numbers used for previous publications, which is most optimal to generate statistically significant results.
Data exclusions	No data were excluded for statistical analyses.
Replication	All attempts at replication were successful.
Randomization	The samples/cells were randomized to be examined. For in vivo mouse xenograft experiments, the mice were randomly grouped prior to the treatments.
Blinding	Blinding was not relevant to the study because all cells/samples were analyzed in the same way.

Reporting for specific materials, systems and methods

We require information from authors about some types of materials, experimental systems and methods used in many studies. Here, indicate whether each material, system or method listed is relevant to your study. If you are not sure if a list item applies to your research, read the appropriate section before selecting a response.

Materials & experimental systems

n/a	Involved in the study	
☐	☒	Antibodies
☐	☒	Eukaryotic cell lines
☒	☐	Palaeontology
☐	☒	Animals and other organisms
☒	☐	Human research participants
☒	☐	Clinical data

Methods

n/a	Involved in the study	
☒	☐	ChIP-seq
☐	☒	Flow cytometry
☒	☐	MRI-based neuroimaging

Antibodies

Antibodies used	Bcl-XL (clone 54H6) (2764s; Cell Signaling, Danvers, MA) ; Bcl-2 (C124) (M0887; Dako, Agilent) ; phospho-histone γ-H2AX (clone JBW301, EMD Millipore, Temecula, CA); 53BP1 (clone 305, Novus Biologicals, Littleton); AnnexinV (563973, BD Biosciences, San Jose, CA)
Validation	Antibody validation was deferred to the manufacturers and was supported by multiple publications.

Eukaryotic cell lines

Policy information about cell lines

Cell line source(s)	The four human HGSOC cell lines used, OV1369(R2), OV90, OV4453 and OV1946, were derived in our laboratory from the ascites of patients diagnosed with HGSOC. The MDA-MB-231 breast cancer cell line was a gift from the laboratory of Dr. John Stagg (CRCHUM, Canada) which was purchased from ATCC.
Authentication	The four human HGSOC cell lines OV1369(R2), OV90, OV4453 and OV1946, were authenticated by Short Tandem Repeat (STR) profiling
Mycoplasma contamination	All cell lines were tested negative for mycoplasma with IDEXX BioAnalytics (Columbia, MO65201)
Commonly misidentified lines (See ICLAC register)	None of the cell lines used in this study are commonly misidentified lines.

Animals and other organisms

Policy information about studies involving animals; ARRIVE guidelines recommended for reporting animal research

Laboratory animals	NRG mice (NOD-Rag1null IL2rgnull, NOD rag gamma) were obtained from the Jackson laboratory (Bar Harbor, ME). All experiments were carried out with 6-week-old females.
Wild animals	The study did not involve wild animals.

Field-collected samples	The study did not involve samples collected from the field.
Ethics oversight	All research involving animals was complied with protocols approved by institutional committee on animal care (CIPA) of the centre de recherche du centre hospitalier de l'université de Montreal (CR-CHUM)

Note that full information on the approval of the study protocol must also be provided in the manuscript.

Flow Cytometry

Plots

Confirm that:

☒ The axis labels state the marker and fluorochrome used (e.g. CD4-FITC).

☒ The axis scales are clearly visible. Include numbers along axes only for bottom left plot of group (a 'group' is an analysis of identical markers).

☒ All plots are contour plots with outliers or pseudocolor plots.

☒ A numerical value for number of cells or percentage (with statistics) is provided.

Methodology

Sample preparation	cell lines
Instrument	BD LSRFortessa
Software	Flow-Jo, Diva
Cell population abundance	A minimum of 30000 cells in the final population
Gating strategy	We removed element under 50 FSC and under 50 SSC which are considered as debris. Under FSC-H/FSC-W we gate the low population to eliminated doublet.

☒ Tick this box to confirm that a figure exemplifying the gating strategy is provided in the Supplementary Information.
